# Network architecture strongly influences the fluid flow pattern through the lacunocanalicular network in human osteons

**DOI:** 10.1007/s10237-019-01250-1

**Published:** 2019-11-28

**Authors:** Alexander F. van Tol, A. Roschger, F. Repp, J. Chen, P. Roschger, A. Berzlanovich, G. M. Gruber, P. Fratzl, Richard Weinkamer

**Affiliations:** 1 Department of Biomaterials, Max Planck Institute of Colloids and Interfaces, 14476 Potsdam, Germany; 2 Berlin‐Brandenburg School of Regenerative Therapies (BSRT), Föhrer Str. 15, 13353 Berlin, Germany; 3 Ludwig Boltzmann Institute of Osteology at the Hanusch Hospital of WGKK and AUVA Trauma Centre Meidling, 1st Medical Department, Hanusch Hospital, Heinrich Collin Str. 30, 1140 Vienna, Austria; 4 College of Engineering, Mathematics, and Physical Science, University of Exeter, Exeter, EX4 4QF UK; 5 Center of Forensic Science, Medical University of Vienna, Sensengasse 2, 1090 Vienna, Austria; 6 Department of Anatomy, Center for Anatomy and Cell Biology, Medical University of Vienna, 1090 Vienna, Austria; 7 Chemistry and Physics of Materials, Paris Lodron University of Salzburg, Jakrob-Haringer Straße 2a, 5020 Salzburg, Austria

**Keywords:** Osteocyte, Lacunocanalicular network, Human osteon, Fluid flow, Lacuna, Canaliculi

## Abstract

A popular hypothesis explains the mechanosensitivity of bone due to osteocytes sensing the load-induced flow of interstitial fluid squeezed through the lacunocanalicular network (LCN). However, the way in which the intricate structure of the LCN influences fluid flow through the network is largely unexplored. We therefore aimed to quantify fluid flow through real LCNs from human osteons using a combination of experimental and computational techniques. Bone samples were stained with rhodamine to image the LCN with 3D confocal microscopy. Image analysis was then performed to convert image stacks into mathematical network structures, in order to estimate the intrinsic permeability of the osteons as well as the load-induced fluid flow using hydraulic circuit theory. Fluid flow was studied in both ordinary osteons with a rather homogeneous LCN as well as a frequent subtype of osteons—so-called osteon-in-osteons—which are characterized by a ring-like zone of low network connectivity between the inner and the outer parts of these osteons. We analyzed 8 ordinary osteons and 9 osteon-in-osteons from the femur midshaft of a 57-year-old woman without any known disease. While the intrinsic permeability was 2.7 times smaller in osteon-in-osteons compared to ordinary osteons, the load-induced fluid velocity was 2.3 times higher. This increased fluid velocity in osteon-in-osteons can be explained by the longer path length, needed to cross the osteon from the cement line to the Haversian canal, including more fluid-filled lacunae and canaliculi. This explanation was corroborated by the observation that a purely structural parameter—the mean path length to the Haversian canal—is an excellent predictor for the average fluid flow velocity. We conclude that osteon-in-osteons may be particularly significant contributors to the mechanosensitivity of cortical bone, due to the higher fluid flow in this type of osteons.

**Electronic supplementary material:**

The online version of this article (10.1007/s10237-019-01250-1) contains supplementary material, which is available to authorized users.

## Introduction

In living bone, osteocytes form a highly organized cell network structure which is deeply embedded within the mineralized bone matrix. Their cell bodies occupy ellipsoidal lacunae, while their long cell processes run within about 300 nm-wide interconnecting canaliculi. Together the lacunae and canaliculi are referred to as the lacunocanalicular network (LCN), which contributes to roughly 2% to the overall porosity of bone (Cardoso et al. 2013). The multiple functions of osteocytes assign them a key role in the maintenance of bone health (Bonewald 2011). An obvious function is an efficient transport and signaling; the transport of nutrients and waste products between blood supply and bone, and the communication with other cells at bone surfaces (Fritton and Weinbaum 2009; Piekarski and Munro 1977). The large surface area of the LCN (Buenzli and Sims 2015) is used to provide access to the mineral reservoir of bone. Osteocytic osteolysis and perilacunar/pericanalicular remodeling refer to processes in which the osteocytes actively change the surrounding bone matrix (Roschger et al. 2019; Teti and Zallone 2009; Tsourdi et al. 2018). Furthermore, osteocyte death can cause local hypermineralization and intra-lacunocanalicular calcification (micropetrosis) (Busse et al. 2010; Frost 1960; Milovanovic et al. 2017; Repp et al. 2017b). Most important for the current study is the osteocytes’ ability to orchestrate bone remodeling. Two main hypotheses, which are not necessarily mutually exclusive, were proposed to explain the mechanism of biophysical stimulation of osteocytes. Microdamage can interrupt cell processes and lead to osteocyte apoptosis and subsequent triggering of bone remodeling (Burr et al. 1985; Verborgt et al. 2000). Alternatively, load-induced flow of interstitial bone fluid throughout the LCN and the resulting shear forces on the cell surface are regarded as mechanical stimulus that can be sensed by osteocytes (Weinbaum et al. 1994). The latter hypothesis is supported by in vitro studies which demonstrated that osteocytes are particularly sensitive to shear stresses in the range from 0.4 to 2 Pa (Jacobs et al. 2010; Klein-Nulend et al. 1995) and that their cell processes are more sensitive than the cell bodies (Adachi et al. 2009).

At the (peri)cellular level, mechanosensorial mechanisms (such as the glycocalyx (Burra et al. 2010), integrins (Geoghegan et al. 2019), primary cilia (Nguyen and Jacobs 2013; Vaughan et al. 2015), stretch-activated ion channels and G-protein coupled receptors) play an important role in the ability of osteocytes to sense mechanical stimuli (Bakker et al. 2001; Jacobs et al. 2010; Uda et al. 2017). For the detection mechanism the importance of the glycocalyx with its tethering fibers was stressed, which can transmit drag forces caused by the fluid flow onto the cell process (Burra et al. 2010). More specifically, it has been suggested that the cell process is attached by integrins to canalicular projections, which are at infrequent, discrete locations along the canalicular wall (Wang et al. 2007). Downstream cellular signaling (e.g., via the Wnt pathway) further processes the stimuli, eventually leading to paracrine and endocrine signaling (e.g., sclerostin, MEPE, OPG, PGE2, NO, IGF-1) to regulate tissue and organ level mechanoresponses (Bakker et al. 2001; Jacobs et al. 2010; Uda et al. 2017).

Even if the interplay between different mechanosensing pathways within cells and between various cell types is not yet fully understood, it is generally accepted that, at the tissue level, the LCN is central for mechanosensing and mechanotransduction. This is evidenced in aging, where the mechanoresponsiveness of bone decreases likely due to changes in the mechanotransduction pathways and LCN morphology (Hemmatian et al. 2017).

In this study, a combination of experimental and computational methods is used to assess the permeability of osteonal bone and the load-induced fluid flow through the LCN in human osteons. An osteon is a fundamental building block of compact bone roughly cylindrical in shape with a Haversian canal in its center. At the inner surface of the osteon, canaliculi enter into the space of the Haversian canal. Whereas many canaliculi from within the osteon connect to the inner Haversian canal, the outer surface is hydrodynamically sealed by the so-called cement line, such that only very few canaliculi pass through this surface (Curtis et al. 1985; Milovanovic et al. 2013). For our work it is important that a distinction between osteons has strong implications on the topology of the LCN. As early as 1853, a particular kind of osteon was described, in which a large osteon nests a smaller second osteon in its center (Tomes and De Morgan 1853). In such osteons the connectivity of the canalicular network between the inner and outer osteon is strongly reduced. The term type II osteon is commonly used in the literature to distinguish them from “ordinary” osteons, termed type I (Andreasen et al. 2018; Arhatari et al. 2011; Ericksen 1991; Maggiano et al. 2016). Type II osteons should per definition have two concentric cement lines (Ericksen 1991). However, in practice it is not an easy task to determine whether a cement line around the inner osteon is actually present (Raguin and Streeter 2018). In a recent anthropological bone study, the term “osteon with a secondary resting line” was used (Tjelldén et al. 2018); however, we prefer to refer to these osteons with the suggestive term “osteon-in-osteon” (Redelstorff et al. 2014). The frequency of such osteon-in-osteons is above 10% in human adults, becoming more common at older age and after periods of hunger (Ericksen 1991; Yoshino et al. 1994). The role of this osteon type and the implication of its unusual LCN topology on mechanosensation are largely unknown.

The fluid flow through the LCN was experimentally studied using tracer experiments, demonstrating that mechanical loading enhanced transport of tracers compared to diffusion (Tate et al. 1998; Zhou et al. 2008). Load-induced fluid velocities of 24–84 µm/s were estimated indirectly from real-time tracer experiments in vivo (Price et al. 2011; Zhou et al. 2008). An excellent tool to complement tracer experiments is the computational approach. With computer simulations the spatially heterogeneous pattern of mechanical strains in the tissues and the load-induced fluid flow within the canaliculi can be studied. In poroelastic models bone is characterized as a material with open liquid-filled porosity (Cowin 1999; Piekarski and Munro 1977). Poroelastic models predicted that strains ranging between 1000 and 3000 µε in osteons lead to fluid shear stresses from 0.8 to 3 Pa. The description is mesoscopic, i.e., the architecture of the vascular and canalicular network is not considered in detail, but only subsumed into a (anisotropic) porous material. Application of poroelasticity to osteons showed that a permeability gradient from the cement line to the Haversian canal strongly influences the fluid pressure, but has only a marginal effect on the fluid flow velocity (Remond et al. 2008). Incorporating micro-CT image information into a poroelastic finite element model of cortical bone of rats demonstrated that the vascular porosity has a major influence on interstitial fluid flow (Gatti et al. 2018).

Detailed images of an osteocyte lacuna and its emerging canaliculi obtained by confocal microscopy (Verbruggen et al. 2012) or synchrotron X-ray phase nano-tomography (Varga et al. 2015) were used to predict in situ local deformations around and in osteocytes. Three different domains were distinguished: the osteocyte, the mineralized extracellular matrix and the pericellular matrix (or glycocalyx) within the space between cell and extracellular matrix (ECM) (Cowin and Cardoso 2015; Sansalone et al. 2013; Thompson et al. 2011; Wijeratne et al. 2016). The complex geometry of the lacunocanalicular network and the resulting need to use sophisticated computational fluid dynamics (CFD) methods make calculations computationally costly, leading to rather small studied specimen volumes. Employing fluid–structure interaction techniques, the highest shear stresses on the cell membrane were found along the cell processes (about 12 Pa) and not on the cell bodies (Verbruggen et al. 2014). Based on the image of a single canaliculus obtained by ultrahigh-voltage electron microcopy tomography, numerical analysis with the lattice Boltzmann method showed that fluid flow was laminar without any vortices, despite significant roughness of the canaliculus wall (Kamioka et al. 2012). On a larger length scale, bone volumes include tens of thousands of canaliculi and the intricate topology of the LCN with its spatially varying density and connectivity is a crucial influencing factor of the fluid flow through the network. Up to now, studies of the flow through the canalicular network were limited to highly idealized network topologies like one-dimensional arrangements of lacunae and canaliculi (Kufahl and Saha 1990), periodically ordered networks (Gururaja et al. 2005) or networks with randomly distributed canaliculi uniform in geometry and tortuosity (defined as the ratio of the effective flow path length to the length of a straight connection) (Anderson et al. 2008; Lemaire et al. 2012; Mishra and Tate 2003; Steck and Tate 2005; Tate 2007).

The aim of the present study is to assess the fluid flow within a realistically described canalicular network architecture in different types of human osteons. We focus on osteons for three specific reasons. First, the hydrodynamic boundary conditions are rather clear with a virtually impermeable cement line as outer boundary and the low-pressure reservoir of the Haversian canal as the inner boundary. Second, in combining staining methods with confocal laser scanning microscopy (CLSM), we were able to image the network architecture of the LCN in three dimensions and macroscopic volumes by previously described methodology (Kamioka et al. 2001; Kerschnitzki et al. 2011a). Specifically, the architecture of the LCN was transferred to a network structure consisting of edges (i.e., the canaliculi) and nodes (i.e., lacunae or intersection points between canaliculi) (Repp et al. 2017b). Third, we compared the canalicular networks in ordinary osteons to those in osteon-in-osteons. Our hypothesis is that such strong modifications of the LCN architecture influence the resulting fluid flow pattern in the canalicular network. The obtained results have to be discussed in terms of the relative contributions of different osteon types to the overall mechanosensitivity of bone.

## Materials and methods

### Sample preparation and rhodamine staining

**Fig. 1:**
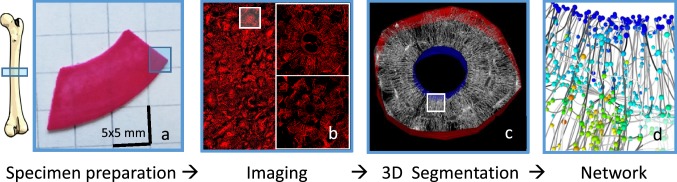
Overview of the experimental workflow from left to right: **a** A section was cut from the midshaft of a human femur and stained with rhodamine before embedding. **b** A 2D overview image of whole specimen was made using confocal laser scanning microscopy (CLSM) in order to identify ordinary osteons and osteon-in-osteons. 3D image stacks of the selected osteons were taken using CLSM. **c** The cement line (red 3D surface) and Haversian canal (blue 3D surface) in each osteon were marked as boundaries for the computational models. Both surfaces appear as areas and not as lines due to the considered depth of the image. **d** The 3D CLSM images were converted into networks consisting of edges (lines representing the canaliculi) and nodes (spheres where canaliculi intersect). A fluid flow analysis was then performed on these networks. Colors of spheres in **d** represent the node pressure, while the darkness of the lines represents the canalicular fluid flow velocity

A necropsy sample of the femoral midshaft of a 57-year-old woman with no known bone-related disease was used for this study. The sample was provided by the Department of Forensic Medicine and the Department of Anatomy of the Medical University of Vienna. The study was performed in accordance with the ethics commission board of this institution (EK no. 1757/2013). Immediately after dissection, bones were frozen and stored at − 20 °C. After unfreezing, the samples were cleaned from soft tissue and a 1-cm-thick piece of the diaphysis was cut perpendicular to the long axis of the bone. From this ring-like cortex, the lateral part was selected for further study (Fig. 1).

Rhodamine staining was used to visualize the LCN without demineralization of the sample following a well-established protocol (Kerschnitzki et al. 2011b). After ethanol dehydration, the bone was placed in a water-free lubricant, diethylene glycol, to prevent water exposure and modification of the bone mineral. The sample was then stained by exposing it under constant motion to a solution of rhodamine-6G in ethanol (0.02% wt) for 24 h. Due to the small size of its molecules (~ 0.4 nm^3^), rhodamine can efficiently diffuse through the entire interconnected LCN and attach to all available surfaces. Since a smooth surface is needed for high confocal image quality, the specimens were then embedded in polymethylmethacrylate (PMMA). The embedded bone was cut in sections with parallel surfaces, sanded with a succession of different grades of abrasive paper and finally polished with diamond powder (Kerschnitzki et al. 2011b). For backscattered electron imaging (qBEI), the sample was coated with carbon to provide a conducting surface.

### Confocal laser scanning microscopy (CSLM) and quantitative backscattered imaging (qBEI)

A Leica TCS SP5 (Wetzlar, Germany) was used to image the 3D LCN of the osteons. A wavelength of 543 nm (HeNe-laser) was used for rhodamine excitation, and the fluorescence signal was measured between 553 and 705 nm with the Airy 1 pinhole of 68 µm. Imaging was done using a 40× oil-immersion lens with a numerical aperture NA = 1.25 (Leica, HCX PL APO 40x NA 1.25 OIL). The side length of the cubic voxel was 300 nm. The imaged volume of a single CLSM image stack was approximately 300 × 300 × 40 µm^3^, which can capture a whole cross section of an osteon. The low imaging depth (40 µm) achieved is due to the limited transparency of mineralized bone. Although the image resolution does not allow assessing the actual diameter of the canaliculi, it is sufficient for an accurate representation of the network topology since distances between canaliculi exceed the resolution (Kerschnitzki et al. 2011b; Milovanovic et al. 2013).

**Fig. 2:**
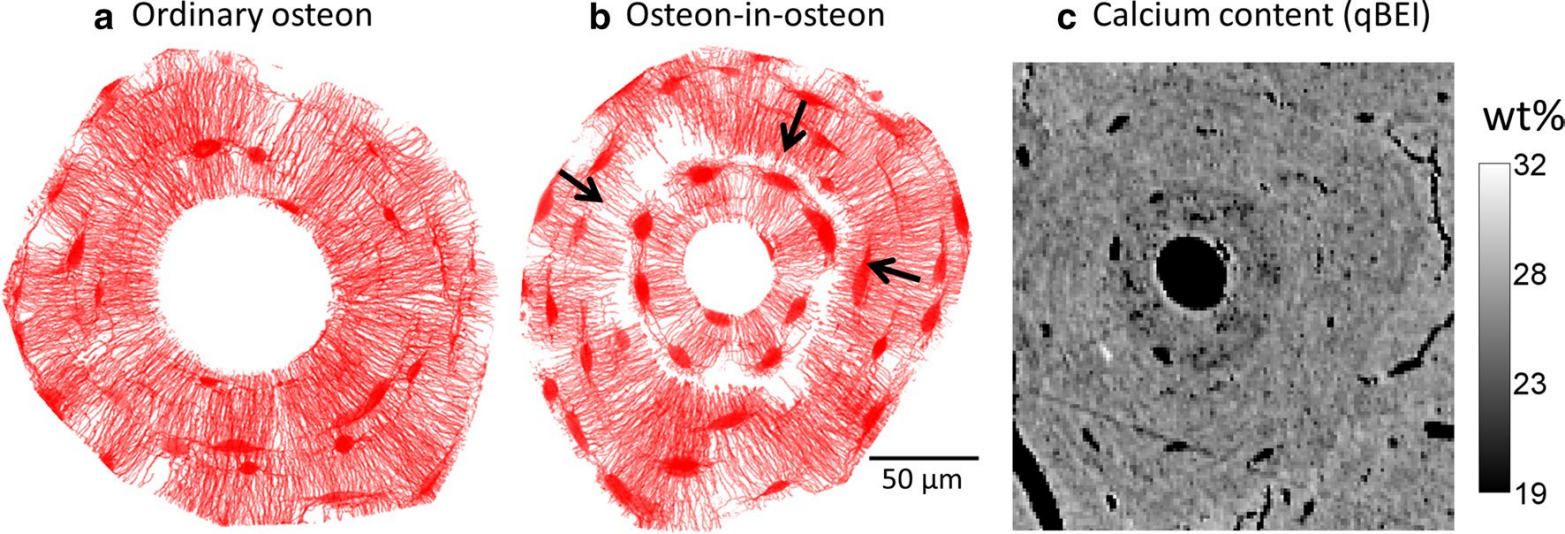
Projections of the 3D lacunocanalicular network of **a** an ordinary osteon and **b** osteon-in-osteon. These binary intensity projections along the imaging depth z-direction were made by taking the thresholded confocal microscopy image stacks with a projection value of 1 (red) if a voxel is part of the LCN and otherwise 0 (white). In the CLSM images osteon-in-osteons can be distinguished from ordinary osteons by an inner ring, which is almost free of canaliculi. The few areas where canaliculi bridge the inner osteon and the outer osteon are indicated with arrows. **c** Quantified backscattered electron image (qBEI) of the same osteon-in-osteon showing quantitatively the local calcium content of the bone. The osteon-in-osteon type is visible by a difference in calcium content between the inner and the outer osteon

Before detailed mapping of osteons, a low-resolution 2D overview image of the full bone specimen was made for the selection of osteons using a low-magnification air lens. In addition, quantitative backscattered electron imaging (qBEI) was used to perform a 2D mapping of the local mineral content of the bone (Roschger et al. 1998). Grayscale images were measured with a digital scanning electron microscope (DSM 962; Zeiss, Oberkochen, Germany) equipped with a four-quadrant semiconductor BE detector. The whole lateral cortex was searched for both types of osteons, ordinary (type I) and osteon-in-osteons (type II). A selection criterion was that the osteon had to be intact, i.e., free of partly remodeled areas and cracks. Osteon-in-osteons could be identified based on the lower mineral content of the inner osteon compared to the outer part and the strongly reduced connectivity of the canalicular network between inner and outer osteons. Osteon-in-osteons were therefore identified using qBEI, as these display two roughly concentric rings of different gray level (Fig. 2c). From the qBEI and CLSM overview images, we initially selected 24 osteons distributed over the whole cortex. After closer visual inspection using CLSM (evaluating their intactness as defined above), 8 ordinary osteons and 9 osteon-in-osteons were selected for detailed investigation.

### Network description of the LCN

The image stack obtained by confocal microscopy was converted into a mathematical network consisting of nodes (i.e., lacunae or points of intersection between canaliculi) and edges (i.e., canaliculi). This multi-step process was performed with the open-source software TINA. A detailed description of each evaluation step is provided on the TINA webpage and in the “Materials and methods” section of previous studies (Repp et al. 2017a, b). What follows is a short summary of the essential steps performed.

First, an adaptive thresholding method was used on the images in the 3D stack to distinguish which parts belong to the LCN and which not. The algorithm subdivides the LCN into lacunae and canaliculi using the much higher “bulkiness” of lacunae compared to canaliculi. Second, the osteon as a region of interest was defined by manually segmenting the outer border of the osteon along the cement line and the inner border along the surface of the Haversian canal. The image was then skeletonized, i.e., voxels representing the canaliculi were evenly removed when possible without changing the connectivity of the network (Weinkamer et al. 2019). The results were strings of voxels marking the center of the imaged canaliculi. This discrete nature of representing canaliculi as a collection of voxels was mitigated by fitting the skeletonized image data by third-order splines. The outcome was a description of the network by the coordinates of its nodes and the connecting edges as a small number of smooth splines. The resulting network was stored as a NetworkX MultiGraph (Schult and Swart 2008).

Parameters that characterize the structure of the network include: (1) the node density, i.e., the number of lacunae and intersection points between canaliculi per unit volume, (2) the canalicular number density, i.e., the number of canaliculi per unit volume, (3) the canalicular length density, Can.Dn, defined as the total length of all canaliculi per unit volume, (4) the node degree as the average number of canaliculi connecting to one node, (5) the weighted node degree, defined as the total length of edges connected to a node, and (6) the average shortest path length between each node *i* and the Haversian canal, calculated using Dijkstra’s algorithm (Dijkstra 1959).

### The lacunocanalicular network as a hydraulic circuit

The calculation of the fluid flow through the LCN was performed in two steps: first (this section), the pressure in the nodes of the network is calculated, and second (next section), the obtained pressure values are combined with the fluid conductivity of a single canaliculi to calculate the fluid flow velocity in each canaliculus.

Kirchhoff’s circuit laws are used to determine the node pressures of the hydraulic circuit. Kirchhoff’s first law (Kirchhoff’s current law) states that the sum of volumetric flow rate at each node must be zero, which reads in matrix form as,

1 $$ \varvec{A}^{T} \varvec{q} = - \varvec{f}, $$

where the edge-node incidence matrix $\varvec{A}$ of the directed network is defined as

2 $$ A^{T}_{ij} = \left\{ {\begin{array}{*{20}l} {   1} \hfill & {{\text{if edge }}j\,   {\text{points towards node }}i} \hfill \\ { - 1} \hfill & {{\text{if edge }}j\,   {\text{points away from node }}i} \hfill \\ {   0} \hfill & {{\text{if edge }}j\,   {\text{is not connected to node }}i} \hfill \\ \end{array} } \right., $$

and $q_{j}$ denotes the volumetric flow rate through the edge $j$ along the direction as defined by $\varvec{A}$ and $f_{i}$ describes the volumetric flow source for node $i$. Note that the introduction of a direction of the network is necessary as reference for the fluid flow, but the sign between a node and a connecting edge in the matrix $\varvec{A}$ is arbitrary. By definition, the volumetric flow rates of $\varvec{f}$ are positive for flow sources and negative for flow sinks. The sum over $\varvec{f}$ must be zero in order to ensure preservation of fluid mass.

The pressure difference within each edge is given by (analogous to Kirchhoff’s voltage law),

3 $$ \Delta \varvec{p} = - \varvec{A p} + \varvec{b}, $$

with $\Delta p_{j}$ being the pressure difference in edge *j*, $p_{i}$ being the pressure at node $i$ and $b_{j}$ describing a fixed pressure source in edge $j$. Darcy’s law relates the pressure and volumetric flow rate within an edge of the network,

4 $$ \varvec{q} = \varvec{C}\Delta \varvec{p} = \varvec{C}\left( { - \varvec{A p} + \varvec{b}} \right), $$

where $\varvec{C}$ is a diagonal matrix with the conductivity $C_{jj}$ of edge $j$ as only nonzero matrix elements.

Combining Eqs. (1) and (4) yields an equation with the pressure in the nodes $\varvec{p}$ as only unknown,

5 $$ \varvec{A}^{T} \varvec{C}\left( { - \varvec{A p} + \varvec{b}} \right) = - \varvec{f}.  $$

Introducing the weighted Laplacian matrix, $\varvec{L} = \varvec{A}^{T} \varvec{CA}$, Eq. (5) can be rewritten as (Grady and Polimeni 2010),

6 $$ \varvec{L p} = \varvec{A}^{T} \varvec{C b} + \varvec{f}. $$

Since only pressure differences are physically relevant, the values of $\varvec{p}$ are defined only up to a constant resulting in a non-invertible matrix $\varvec{L}$. Standard practice to deal with this problem is to select one node as a reference and set the pressure there equal to zero. We assigned the reference status to a special node $i_{0}$, which was introduced to link all edges that connect to the Haversian canal. This corresponds to the assumption that the vascular porosity is a constant low-pressure reservoir (Cowin 1999). Equation (6) is then solved for $\varvec{p}$ using the reduced Laplacian $\varvec{L}_{0}$ defined as the Laplacian matrix $\varvec{L}$ with the $i_{0}$th row and column removed (Grady and Polimeni 2010; Newman 2010). All calculations were performed with Scipy 0.15.1 and Numpy 1.9.2 in Python 2.7 (http://python.org). The matrices $\varvec{A}$ and $\varvec{L}$ were constructed from the TINA networks using NetworkX 1.10. Employing the SciPy 0.15.1 sparse matrix methods, in combination with rearranging the node indices with the reverse Cuthill–McKee algorithm (Cuthill and McKee 1969), allowed for the calculation of the pressure patterns within a whole 40-µm-thick osteon within a couple of seconds.

### Fluid flow in single canaliculi

The entries of the conductivity matrix $\varvec{C}$ are obtained by assessing the fluid flow within a single canaliculus (corresponding to edge $j$ from Sect. 2.4) starting with Darcy’s law for the volumetric flow rate $q_{j}$,

7 $$ \frac{{q_{j} }}{A} = - \frac{{k_{{p,{\text{eff}}}} }}{\mu } \cdot \nabla_{j} p, $$

where $\nabla_{j} p$ denotes the pressure gradient in the axial direction of the canaliculus and $\mu$ the denotes viscosity of the interstitial bone fluid. In estimating the cross-sectional area $A$, it is important to consider that the bone fluid can flow only in the annulus between the cell processes of the osteocyte and the canalicular wall. To estimate the effective permeability, $k_{{p,{\text{eff}}}}$, we follow the approach by Weinbaum et al. (1994) by taking into account, firstly, that a fibrous matrix exists within the annular region between the osteocyte process and the canalicular wall consisting predominantly of proteoglycans which strongly influence the permeability of canaliculi (Cowin and Cardoso 2015; Sansalone et al. 2013; Thompson et al. 2011; Tsay and Weinbaum 1991; Wijeratne et al. 2016). Assuming a two-dimensional square array of fibers, an expression can be obtained which includes only the fiber radius and the fiber spacing as geometric parameters (Tsay and Weinbaum 1991). Secondly, homogenization results in a Brinkman equation, which is solved with no-slip boundary conditions at the canaliculus wall and the surface of the osteocyte process. The resulting numerical values for $k_{{p,{\text{eff}}}}$ and all other model parameters are summarized in Table 1.

**Table 1:** Numerical values of model parameters

Parameter	Value	Description
$\mu$	1.06 × 10^−3^ Pa s	Viscosity of the bone fluid (Cardoso et al. 2013)
$Ca.Rd$	157.5 nm	Radius of the canaliculus (Varga et al. 2015)
$CP.Rd$	73 nm	Radius of the osteocyte process (Buenzli and Sims 2015)
$A$	0.061 µm^2^	Annular cross section between canaliculus and osteocyte process calculated as: $A = \left( {Ca.Rd^{2} - CP.Rd^{2} } \right)\pi$
$k_{{p,{\text{eff}}}}$	1.53 × 10^−17^ m^2^	Permeability of a canaliculus (Weinbaum et al. 1994)
$K$	465 µm^−1^	Shear stress constant (Weinbaum et al. 1994)
$\dot{\epsilon }$	0.015 s^−1^	Volumetric strain rate value corresponds to peak strain rate during exercise (Al Nazer et al. 2012; Lanyon et al. 1975; Milgrom et al. 2002)
$V_{i}^{\text{lacuna}}$	350 µm^3^	Lacunar volume (Carter et al. 2013; Dong et al. 2014)

The combination of Eqs. (4) and (7) using a linear pressure decrease in the canaliculus gives the conductivity,

8 $$ C_{jj} = \frac{{q_{j} }}{{\Delta p_{j} }} = - \frac{{k_{{p,{\text{eff}}}} }}{\mu }\frac{A}{{l_{j} }}, $$

with $l_{j}$ being the length of the canaliculus. The average velocity of the bone fluid in the canaliculus, $v_{j}$, is then

9 $$ v_{j} = \frac{{q_{j} }}{A} = - \frac{{k_{{p,{\text{eff}}}} }}{\mu }\frac{\Delta p}{{l_{j} }}. $$

Assuming a no-slip boundary condition, the shear stress *τ* on the cell process membrane can be obtained from the velocity gradient at the cell membrane, as given by (Weinbaum et al. 1994; You et al. 2001),

10 $$ \tau = \left. {\mu \frac{\partial u}{\partial r}} \right|_{r = CP.Rd} = \mu \cdot K \cdot v , $$

where $u\left( r \right)$ denotes the cylindrically symmetric velocity profile in the annulus region between cell process and canaliculus wall.

### Load-induced fluid flow—boundary conditions

The network analysis of 2.4 provides the incidence matrix $\varvec{A}$ and the length of the canaliculi $l_{j}$ so that using Eq. (8) the conductivity matrix $\varvec{C}$ can be calculated. To calculate the pressure in each node of the LCN using Eq. (6), the fluid flow source in each node $\varvec{f}$ and the pressure source in each edge $\varvec{b}$ of the network have to be defined as boundary conditions. Two approaches were used.

#### Approach 1: Intrinsic osteon permeability

The purpose of this approach is to determine the intrinsic permeability of the osteon. Therefore, a fixed pressure difference between the outside (cement line) and the inside of the osteon (Haversian canal) is applied and the resulting fluid flow through the LCN into the reservoir of the Haversian canal is studied. The intrinsic permeability describes how the measured network structure in the osteon resists the fluid flow through the LCN. Due to the connectivity of the LCN and the canalicular tortuosity (both of a single canaliculus and the interconnection of many canaliculi to cross the osteon from the cement line to the Haversian canal), the intrinsic permeability of the osteon is lower than the effective permeability of a canaliculus,$k_{p,{\rm eff}}$. As boundary conditions a high pressure is assumed at the outer cement line, while the pressure in the Haversian canal is used as a reference pressure set to zero,

11 $$ \varvec{b} = \varvec{A}\left\{ {\begin{array}{*{20}l} { 13 {\text{kPa}}} \hfill & {\text{for all nodes at the cement line}} \hfill \\ {   0 {\text{kPa}}} \hfill & {\text{for all the other nodes}} \hfill \\ \end{array} } \right.\,{\text{and}}\quad\,{\bf f} = 0. $$

The top and bottom surfaces of the osteon are sealed off. Multiplication with the incidence matrix $\varvec{A}$ is necessary since $\varvec{b}$ is defined on edges. The numerical value of $\Delta p_{\text{osteon}}$ = 13 kPa was chosen to have a similar range of pressure occurring in the network as in approach 2. The intrinsic permeability $k_{\text{osteon}}$ can then be calculated from the resulting average fluid flow velocity, $\bar{v}_{\text{osteon}}$, and osteon wall thickness, $\Delta R$:

12 $$ k_{\text{osteon}} = \bar{v}_{\text{osteon}} \cdot \mu \cdot \frac{{{{\Delta }}R}}{{{{\Delta }}p_{\text{osteon}} }}, $$

13 $$ \hbox{where}\quad\bar{v}_{\text{osteon}} = \frac{{\sum v_{j} \cdot l_{j} }}{{\sum l_{j} }} $$

#### Approach 2: Deformation-induced forced fluid flow

The purpose of this approach is to model how deformations of osteons force bone fluid through the LCN to the Haversian canal. The osteon and its LCN are viewed as a virtually sealed off building block of cortical bone. For the simulations presented below, the cement line was modeled as an impermeable boundary. Canaliculi stop at the cement line and, therefore, constitute a dead end for the fluid flow. Upon loading, the bone, including the osteon, is deformed at a certain strain rate. The deformation is assumed to be homogeneous in the osteon, and the pericellular space between cell and ECM is assumed to be filled with fluid. The reduction in pore volume, therefore, squeezes the fluid toward the openings of low pressure, i.e., toward the Haversian canal. Both lacunae and canaliculi have fluid-filled pore volumes which contribute to the load-induced fluid flow. According to our model, each node in the network acts as a source of fluid, where the value of $\varvec{f}$ depends on the volumetric strain rate $\dot{\epsilon }$ and the volume of the node. Strain rate is likely the main contributor to fluid flow velocity in the LCN (Goulet et al. 2009; Remond et al. 2008; Wu et al. 2016). The value for the strain rate $\dot{\epsilon }$ was chosen following experiments with strain gauges on the surface of the human tibia (Al Nazer et al. 2012). Peak strain rates that occur during exercise were chosen, as they have been shown to induce an anabolic response in the bone (Lewis et al. 2017).

A canaliculus is always shared between two nodes; therefore, the node volume is calculated as half the volume of the canaliculi connecting to the node. In case the node represents an osteocyte lacuna, a constant lacunar volume $V_{i}^{\text{lacuna}}$ is added. The boundary conditions are, therefore,

14 $$ \varvec{f} = \left\{ {\begin{array}{*{20}l} { - \dot{\epsilon } \cdot V^{\text{OLCN}}  } \hfill & {\text{node representing the Haversian canal}} \hfill \\ { + \dot{\epsilon } \cdot \left( {\frac{{{ \deg }_{i} }}{2} \cdot A + V_{i}^{\text{lacuna}} } \right)} \hfill & {\text{for all lacunae}} \hfill \\ { + \dot{\epsilon } \cdot \frac{{{ \deg }_{i} }}{2} \cdot A} \hfill & {\text{for all other nodes}} \hfill \\ \end{array} } \right.\,{\text{and}}\quad\,{\bf b} = 0, $$

where $\deg_{i}$ is the weighted node degree, i.e., the sum over the length of all canaliculi connecting to node $i$ (Schult and Swart 2008). The bone fluid volume that is squeezed into the Haversian canal is given by the condition that the sum over $\varvec{f}$ has to be zero to comply with the preservation of fluid mass.

### Statistical analysis

Comparison between ordinary osteons and osteon-in-osteons was done using two-tailed Wilcox rank-sum test. *P* values of lower than 0.05 were considered to be significant, and results were reported as mean value ± standard deviation. Simple linear regression analysis was used to find correlation coefficients between fluid flow and structural network parameters. Locally weighted scatterplot smoothing (LOWESS) was used to fit a smooth line through the pressure gradient data (Fig. 4). To calculate the weighting function of LOWESS, data points contained within a span of 10% regression were included. To quantify the angular dependency of the network parameters, osteons were subdivided into 36 segments of 10 degree opening angle around the center of the Haversian canal. Variability was then assessed by the relative standard deviation, i.e., the standard deviation normalized by the average value. All calculations were performed in Python.

## Results

### Structural characterization of osteons and LCN within osteons

As a first step, a structural comparison of the size of the ordinary osteons (*N* = 8) and osteon-in-osteons (*N* = 9) and their corresponding 3D osteocyte lacunocanalicular network (LCN) was performed. Average structural parameters are shown in Table 2. The sizes of the osteons in the two groups were very similar. However, the size of the Haversian canal was smaller in osteon-in-osteons compared to ordinary osteons (*P* < 0.001). Basic parameters characterizing the LCN within the osteon, like number density of nodes (i.e., the number of lacunae and intersection points between canaliculi per volume), number density of canaliculi (i.e., number of canaliculi per volume), node degree (i.e., average number of canaliculi meeting in a node) and weighted node degree, did not show any significant difference depending on osteon type. However, the average shortest path length through the network from each node to the Haversian canal was 80% longer in the osteon-in-osteon group (*P* < 0.01). This difference in the accessibility of the Haversian canal can be explained by looking at the networks of the two osteon types (see two representative examples in Fig. 2). A predominant alignment of canaliculi in the radial direction toward the Haversian canal can be observed in both osteon types. Both osteons show some network-free regions. The most salient difference is that in the osteon-in-osteon there is a ring visible where the network density is much lower. This local drop in network density divides the osteon into two parts which are referred to as inner and outer osteon. The network connections between the inner and outer osteon occur only via a few bridges as indicated by the arrows in Fig. 2. As a consequence, to connect parts of the network from the outer osteon to the Haversian canal, longer detours are needed to reach one of the bridges to overcome the gap between the inner and outer osteon. Figure 2b shows the same osteon-in-osteon imaged by electron microscopy in backscattered mode (qBEI). Darker gray levels denote lower mineral content and are commonly associated with younger tissue (Tjelldén et al. 2018). Two gray levels are visible within this osteon, with the outer osteon being more mineralized than the inner one.

**Table 2:** Structural parameters for each osteon-type group (significant difference *P < 0.01, **P < 0.001)

	Ordinary osteons	Osteon-in-osteons
Osteon parameters		
On.Rd	104 ± 22 µm	103 ± 13 µm
HCa.Rd	36 ± 6 µm	22 ± 5 µm **
Evaluated.BV	1.2 × 10^6^ ± 5.6 × 10^5^ µm^3^	1.3 × 10^6^ ± 3.0 × 10^5^ µm^3^
LCN parameters		
Number density of nodes	0.015 ± 0.002 1/µm^3^	0.014 ± 0.003 1/µm^3^
Number density of canaliculi	0.021 ± 0.003 1/µm^3^	0.019 ± 0.004 1/µm^3^
Length density of canaliculi	0.081 ± 0.008 µm/µm^3^	0.072 ± 0.009 µm/µm^3^
Node degree	3.62 ± 0.08	3.65 ± 0.04
Weighted node degree	11.9 ± 0.76 µm	11.3 ± 0.90 µm
Average shortest path length	50.0 ± 11.5 µm	92.8 ± 32.3 µm *

### Fluid pressure patterns in the LCN

**Fig. 3:**
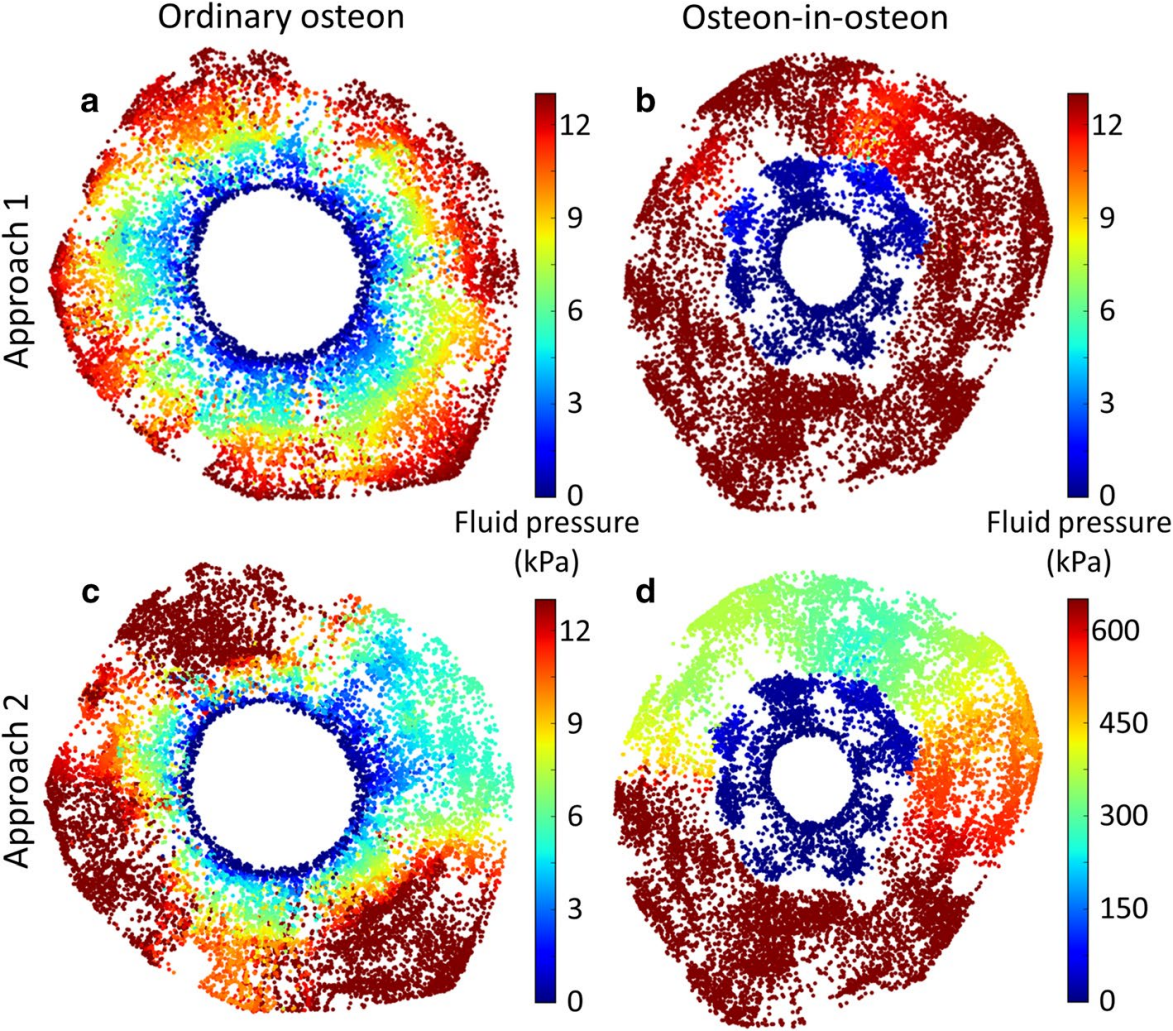
Pressure pattern images were made by plotting color-coded spheres at the location of each node of the network for a representative ordinary osteon (left) and osteon-in-osteon (right) (same osteons as in Fig. 2). The fluid flow was modeled with two approaches. Approach 1) A pressure difference of 13 kPa between cement line and Haversian canal was applied to an ordinary osteon (**a**) and an osteon-in-osteon (**b**). Approach 2) Fluid flow was forced out of the ordinary osteon (**c**) and osteon-in-osteon (**d**) as water is squeezed out of a steadily compressed sponge (i.e., constant homogeneous strain rate). The difference in pressure patterns between the two osteon types is a direct result of a difference in LCN topology. The much higher pressure in **d**) (note the different color scale) is partly caused by the much lower permeability of this osteon-in-osteon

The two osteons of different types introduced in Fig. 2 were then used to demonstrate differences in the fluid flow properties of the canalicular network when subject to different modeling approaches. Figure 3 depicts the pressure pattern in the two osteons when using our two modeling approaches: (1) a pressure gradient was applied from the cement line to the Haversian canal (Fig. 3a, b); (2) a reduction in the pore space forced the fluid to flow toward the Haversian canal, analogous to squeezing a fluid out of a sponge (Fig. 3c, d).

**Fig. 4:**
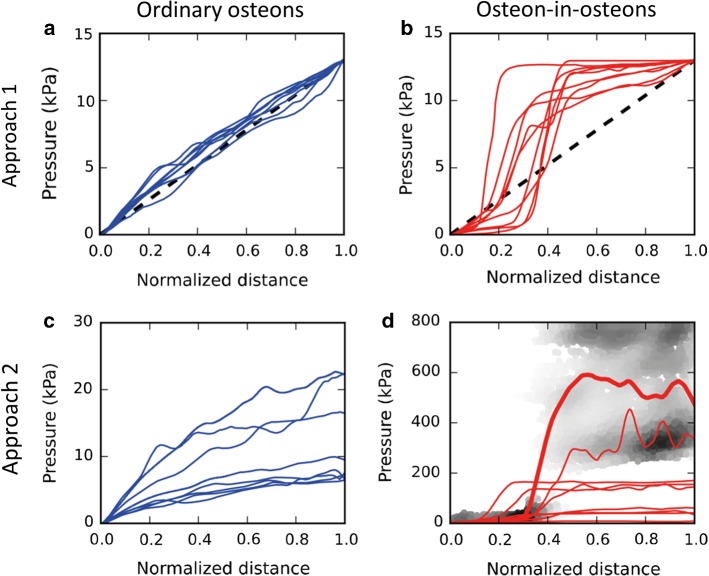
Pressure profiles plotted as a function of the normalized distance (Haversian canal = 0, cement line = 1). The profiles were obtained, firstly, by presenting for each node the values of its position (given by **a** value between 0 and 1 as normalized distance) and its pressure. Secondly, the data of all nodes in this scatter plot (plot not shown) were transformed in the shown profiles by using a local regression algorithm (locally weighted scatterplot smoothing, LOWESS). **a** and **c** Show the pressure profile for all 8 ordinary osteons; **b** and **d** show pressure profiles for all 9 osteon-in-osteons. In a and b the dashed line serves as a reference of a linear decrease of the applied pressure. In the background of d the gray values indicate the density of data points of the scatter plot for the osteon-in-osteon of Fig. 3d. The corresponding LOWESS fit is shown by the thicker red line. This kind of representation was chosen to highlight the spatial heterogeneity of the pressure distribution in this case, which can be only poorly rendered by a LOWESS fit

Approach 1 (Fig. 3a, b) results in pressure patterns which majorly differ depending on the type of osteon. While in the ordinary osteon the pressure drops more or less continuously when moving toward the Haversian canal, the osteon-in-osteon displays a sudden change in pressure exactly between the inner and outer osteon. These very different pressure profiles are best demonstrated when the pressure is plotted against the normalized distance from the Haversian canal to the cement line (Fig. 4a, b). In all osteon-in-osteons the pressure shows a sudden decrease (Fig. 4b). The main difference in the pressure pattern across osteon-in-osteons is the location of the sudden decrease occurs, which is unmistakably related to the transition between the outer to the inner osteon. In all the studied osteon-in-osteons the boundary between the inner and outer osteon was closer to the Haversian canal (i.e., normalized distance < 0.5), indicating that the wall thickness of inner osteons was always smaller compared to that of outer osteons. The pressure profiles in the investigated ordinary osteons are very similar (Fig. 4a) and can be accurately approximated by a linear decrease in pressure (dashed line in Fig. 4a). Deviations from linearity occur near the Haversian canal, where most ordinary osteons show a slightly steeper slope, and close to the cement line, where the slope tends to be shallower.

Modeling with approach 2(Fig. 3c, d) demonstrates that, similar to approach 1, two different ranges of pressure are observed in osteon-in-osteons, one in the inner and one in the outer osteon. However, the pressure patterns are more intricate with approach 2, mainly due to angular dependency as a new feature. The values of the pressure encountered on a radial path from the cement line to the Haversian canal depend strongly on the starting point at the cement line. Using as an example the osteon of Fig. 3c, the pressure gradient is maximal at a direction corresponding to 12 o’clock while rather nearby at around 2 o’clock the pressure gradient is much lower. This heterogeneity of the pressure pattern is more pronounced for the osteon-in-osteons. To quantify the angular dependency of the pressure pattern, the roughly circular osteon was subdivided into 36 sectors with an opening angle of 10 degrees and the average pressure was calculated over all the nodes in each sector together with the relative standard deviation of these 36 mean values. While for model approach 1 the relative standard deviation was only 12% for both ordinary osteons and osteon-in-osteons, it was 44% for ordinary osteons and 53% for osteon-in-osteons for model approach 2.

Another new feature that was introduced by using modeling approach 2 is that the variability of the pressure pattern between the studied osteons is much bigger, particularly in the osteon-in-osteon shown in Fig. 3d. As shown in Fig. 4c, the pressure profiles are nonlinear and differ widely across ordinary osteons. For example, the pressure at the cement line—which is not fixed in model approach 2—varies by more than a factor of three, between 7 and 25 kPa. This high variability is even stronger in osteon-in-osteons (Fig. 4d). In this case, a LOWESS fit provides only a poor rendering of the pressure profile from the Haversian canal to the cement line due to the strong angular dependency of the pressure pattern. The pressure values which are found in osteon-in-osteons are much higher than in ordinary osteons: with approach 2 the average pressure in the osteon-in-osteons is 12 times higher than the average pressure of ordinary osteons.

### Fluid flow patterns in the LCN

**Fig. 5:**
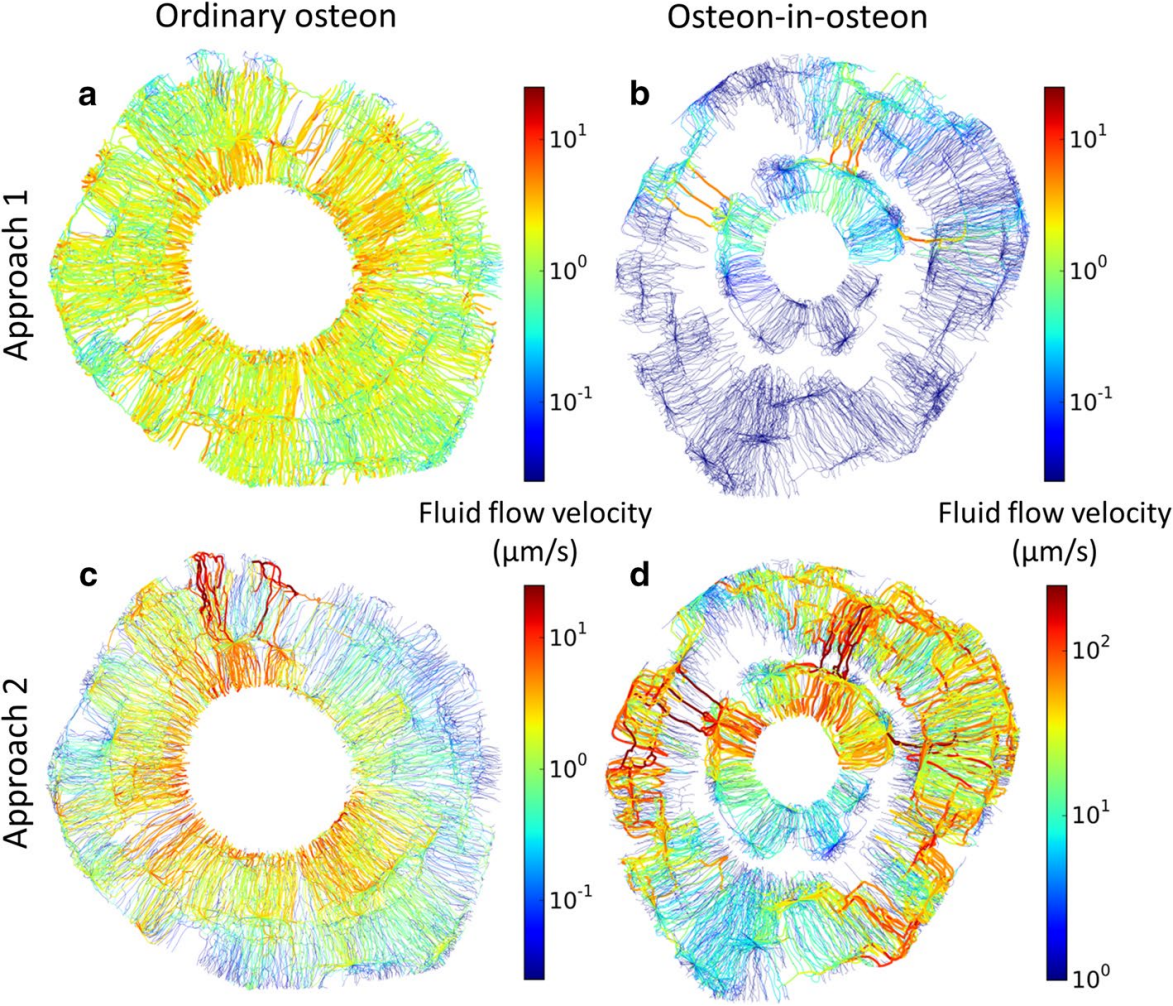
The fluid flow patterns in the lacunocanalicular network are projections of the network in the style of a road map, where in addition to the color code edges with higher fluid flow velocity are rendered thicker. Edges with vanishing fluid flow velocity are not shown. **a** and **b** show the fluid flow patterns resulting from approach 1 in an ordinary osteon and a osteon-in-osteon, respectively; **c** and **d** the resulting fluid flow patterns using approach 2. The difference in fluid flow patterns between the two osteon types is a direct result of a difference in LCN topology

**Fig. 6:**
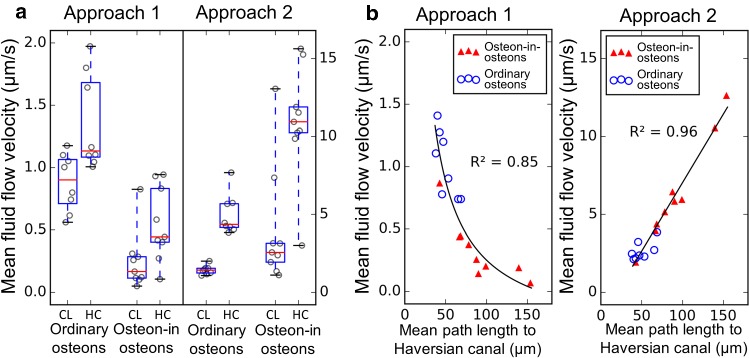
Comparison of average fluid flow velocities. Each data point in the plots represents one osteon. **a** Comparison between fluid flow velocity in the outer half (CL for half close to cement line) and the inner half (HC for half close to Haversian canal) of the ordinary osteons. The box extends from the first to third quartile, the red line shows the median, and the whiskers extend from the box to show the full range of the data. **b** Relationship between average fluid flow velocity and mean path length to the Haversian canal. Different models were used to fit the data (black lines). For approach 1 the average fluid flow velocity is inversely related with the average shortest path length. For approach 2 the fluid flow velocity is linearly related with average shortest path length

The two osteons introduced in Fig. 2 are again used to demonstrate differences in the fluid flow pattern. Comparison of the results from model approach 1 reveals a higher average fluid flow velocity in the ordinary osteon than in the osteon-in-osteon (Fig. 5a, b). In ordinary osteons the fluid flow velocity is higher near the Haversian canal than near the cement line (Figs. 5a, 6a). In osteon-in-osteons the only areas with high fluid flow velocity are the bridges between the inner and outer osteon.

Using Eq. (12) to determine the intrinsic permeability of the osteons, the average permeability of the ordinary osteons is $k_{\text{osteon}} =$ 11 ± 2.2 10^−18^ m^2^ (i.e., 72% of $k_{{p,{\text{eff}}}}$). For osteon-in-osteons this permeability is almost three times lower, $k_{\text{osteon}} =$ 4.1 ± 2.2 10^−18^ m^2^ (i.e., 72% of $k_{{p,{\text{eff}}}}$), *P* value < 0.001.

The load-induced fluid displacement of approach 2 causes the fluid flow in osteon-in-osteons to be concentrated on certain “canalicular highways” (dark red edges in Fig. 5d), which conduct the fluid to the bridges connecting inner and outer osteons. Although in both model approaches the fluid flow heterogeneity is higher in osteon-in-osteons, the consequences for the fluid velocity averaged over the whole osteon are exactly the opposite. Approach 1 (Fig. 6a, left) results in an average fluid velocity which is in the osteon-in-osteon 3.1 times reduced compared to ordinary osteons (*P* < 0.001), while this velocity is 2.3 times increased in osteon-in-osteons for approach 2 (*P* < 0.01) (Fig. 6a, right).

A feature independent of the osteon type and the model approach is that average fluid velocities are higher close to the Haversian canal compared to close to the cement line (Fig. 6a). Figure 6b now relates a purely structural network parameter—the mean path length to the Haversian canal—with the average fluid velocity. For both approaches, there is a clear relationship between these two quantities, although of opposite character. For approach 2, the average velocity in the network can be accurately predicted from the average shortest path length based on a linear relationship between these two quantities. In contrast, approach 1 results in an inverse relationship such that networks with a large average mean path length to the Haversian canal have on average a lower flow velocity.

**Fig. 7:**
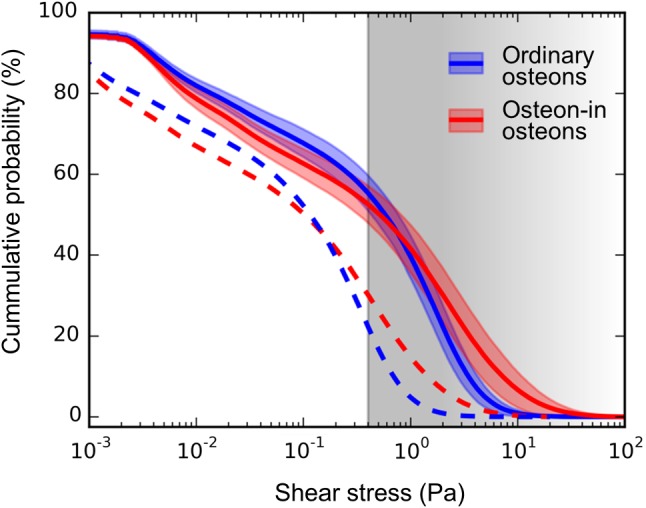
The cumulative probability distributions of fluid shear stresses at the cell process surfaces in the canaliculi are plotted for ordinary osteons (blue) and osteon-in-osteons (red) for the case of strenuous exercise (solid lines) and normal daily activities (dashed lines). Fluid flow velocities were calculated using model approach 2 and from the velocities shear forces were obtained using Eq. (10). All lines show the mean percentage of canaliculi with a shear stress larger than the value on the x-axis. The 99% confidence intervals of the mean are shown in the faintly colored bands around the lines. Confidence intervals are the same for both cases and are, therefore, omitted for the dashed lines to improve readability. The gray area above 0.4 Pa illustrates the range of shear stresses where osteocytes showed osteogenic responses to fluid shear stress in in vitro experiments (Bacabac et al. 2004; Bakker et al. 2001; Jacobs et al. 2010; Klein-Nulend et al. 1995)

To put our results now in the context of the suggested mechanosensitive response of osteocytes to shear forces, Eq. (10) was used to transform velocities into shear forces for approach 2. The cumulative probability distributions of shear stresses in Fig. 7 reveal which percentage of cell processes in the canaliculi is exposed to shear forces larger than the value plotted on the x-axis. For comparison, the shear forces above 0.4 which were demonstrated through in vitro experiments to trigger osteogenic responses in osteocyte-like cells (Bacabac et al. 2004; Bakker et al. 2001; Jacobs et al. 2010; Klein-Nulend et al. 1995) is shaded in gray. The cumulative probability distributions for ordinary osteons and osteon-in-osteons intersect each other at a shear stress of 0.8 Pa and a probability of 45%. This means that, in osteon-in-osteons, there is a higher amount of canaliculi subject to shear stresses above 0.8 Pa. Decreasing the strain rate from 0.015 to 0.0015 s^−1^ (i.e., simulation of walking instead of running) shifts the intersection to 0.08 Pa. Here the percentage of canaliculi which are stimulated with shear stress larger than 0.8 Pa value are 6.6-times more numerous in osteon-in-osteons than in ordinary osteons (9.3% and 1.4%, respectively).

## Discussion

The assessment of bone’s mechanosensitivity and mechanoresponse is the crucial aspect of understanding bone health. Using in vivo microcomputed tomography on mice, only recently have first steps been made toward a more quantitative description of Wolff’s law (Birkhold et al. 2015; Lambers et al. 2011). With age the mechanoresponse was shown to lose rigor (Razi et al. 2015). Changes in the responsiveness of bone are very often addressed on the cellular and subcellular level by elucidating signaling pathways from the cell membrane to the nucleus and back (Chen et al. 2010; Jacobs et al. 2010). However, a general characteristic of biological sensing is the integration of multiple length and time scales to amplify the stimulus signal and to improve its fidelity. For bone’s fluid flow-based mechanosensitivity hypothesis, this implies not to focus exclusively on osteocytes and their interaction with each other, but also to consider the flow of the interstitial fluid through the osteocyte lacunocanalicular network (LCN) and the modification of this flow depending on the topology of the LCN.

The current work combines two length scales in the study of fluid flow through the canalicular network: The overall architecture of the LCN with its roughly 25000 canaliculi within the imaged volume of osteonal bone and the fluid flow within the canaliculi. The permeability of the canaliculi is reduced due to the presence of cell processes in their center and a fibrous matrix in the surrounding pericellular space (Cowin and Cardoso 2015; Sansalone et al. 2013; Thompson et al. 2011; Wijeratne et al. 2016). In our analysis, four experimental and computational methods were combined: (1) staining and laser confocal microscopy to image the three-dimensional LCN, (2) image analysis to transfer the image into a mathematical network structure, (3) a description of bone inspired by poroelasticity to model load-induced fluid flow in the LCN and (4) circuit theory based on Kirchhoff’s circuit laws to calculate the fluid velocity in the individual canaliculi of the network.

For the computational analysis of the fluid flow in the canalicular network, we employed two complementary modeling approaches. Model approach 1, with its simple assumption of a pressure gradient between the cement line and the Haversian canal, allows assessing the fluid flow resistance of the entire canalicular network. Similar approaches were used in earlier LCN fluid flow studies (Anderson et al. 2008; Steck and Tate 2005) and have the advantage of yielding an important bone material parameter, its intrinsic permeability, a result which is rather easily interpretable served as a reference for comparisons as well as a guide to build intuition when using the more realistic model approach 2 of load-induced fluid flow. Approach 2 describes how dynamic loading of our bone compresses the bone and the embedded porosity within the bone. Since the pores are liquid-filled and their volume is reduced by the compression, the liquid has to move. In osteons it moves toward the Haversian canal, since there the network is “open” and the liquid can be easily drained to this low-pressure reservoir. In this second approach the LCN porosity therefore acts not only as transport network for the fluid but also as source of fluid that has to be drained via the network. Such a poroelastic description of bone is the preferred model approach of most researchers studying load-induced fluid flow in the LCN (Cardoso et al. 2013; Nguyen et al. 2010; Smit et al. 2002; Weinbaum et al. 1994).

Several studies have explored the effect of an idealized LCN network topology on fluid flow in bone. Not only has the topology of single canaliculi (e.g., tortuosity) been taken into account (Lemaire et al. 2012), but also the influence of a network with idealized connectivity (Anderson et al. 2008; Steck and Tate 2005). New developments in the field of confocal microscopy and image analysis make it now possible to image the topology of LCN networks in macroscopic bone volumes faithfully (Repp et al. 2017b). The use of network data obtained in this way allowed us to study the fluid flow through realistic canalicular networks of full cross sections of human osteons. However, the diameter of canaliculi is too small to be resolved by optical microscopy. The imprecision introduced in our calculations by assuming a homogeneous width of the pericellular space is rather low since the pericellular space between cell process and canaliculus wall is not empty. The fibrous matrix within this space prevents the formation of a parabolic flow profile in the annulus (Weinbaum et al. 1994), so that the effective permeability depends only mildly on the width of the pericellular space (Lemaire et al. 2012).

The opaqueness of the bone limits the imaging depth to about 40 µm. This limitation in the dimensions of the imaging volume should have only minor effects on the results of our study, since the LCN only shows reduced structural heterogeneity in the direction perpendicular to the imaging plane compared to the much more pronounced heterogeneity within the imaging plane, particularly in the radial direction near the Haversian canal. We ensured that the axial directions of the imaged osteons were perpendicular to the imaging plane by only selecting osteons where the Haversian canals appeared circular and straight along the z-axis. Additionally, the selected osteons were not close to Volkmann’s canals and other structural features, which could influence the network topology in the direction perpendicular to the imaging plane. Previous studies could not show substantial differences in LCN structures between individuals (Repp et al. 2017b; Weinkamer et al. 2019). Therefore, the limitation of using one human specimen for all the analysis should have no major impact on the main conclusions of our work. A limitation in approach 2 is that we abstain from an intricate biomechanical description of the loading, but assume a homogeneous strain rate, as this is often found to be a main determinant of fluid flow velocity in the LCN. To assume that the resulting strain is homogeneous is reasonable, since the low porosity does not significantly change the elastic properties of osteons (Yoon and Cowin 2008). In the presented data we neglected differences in the compressibility of the solid and fluid phases, which could limit the values of the pore pressure (Cowin 1999). To investigate this influence, we therefore included a saturation pressure to test the robustness of our results (see Eqs. (15) and (16) in the Supplementary Material). This saturation pressure had no significant influence on the outcomes, even when applied to the osteon-in-osteon which showed the highest fluid pressure (see Supplementary Material for a detailed discussion). Also poroelastic models of osteons showed that a limitation of the pressure only has a limited effect on the fluid flow velocity (Remond et al. 2008; Yoon and Cowin 2008).

Model approach 1 simulates measurements of the intrinsic permeability by directly relating velocity in a canaliculus to the pressure difference between the nodes delimitating the canaliculus (Eq. 9). Consequently, the roughly linear decrease of the pressure from the cement line to the Haversian canal translates into a roughly homogeneous fluid velocity in ordinary osteons (Fig. 5a). For an idealized canalicular network, where the canaliculi just run straight from the cement line to the Haversian canal, the fluid velocity would be constant in all canaliculi. Realistic osteon networks show a slight tree-like network topology, where the number of parallel canaliculi increases with distance from the center of the osteon (Repp et al. 2017b; Roschger et al. 2019). Consequently, the resistance of the network is higher close to the Haversian canal resulting in a larger than average drop in pressure (Fig. 4a) with a slightly increased fluid flow velocity (Fig. 5a). For osteon-in-osteons such a local increase in the network resistance is extremely pronounced at the interface between outer and inner osteons. The strong reduction in network density with only a few bridges connecting the two parts of the osteon results in a drop of pressure in this location (Fig. 4b). Due to overall higher network resistance, the average fluid flow is reduced in osteon-in-osteons using approach 1 and only in the bridges connecting outer and inner osteons higher velocities are obtained (Fig. 5b).

Use of approach 2 not only results in pressure patterns that are quantitatively different from approach 1, but also fluid velocity patterns which are qualitatively different. The flow pattern includes very high fluid velocities and a strong spatial heterogeneity. Following only canaliculi with high fluid velocity (marked in red in Fig. 5d), paths can be found that connect the cement line to the Haversian canal. However, these paths are not straight and rather lengthy, due to the requirement that they have to pass via the few bridges connecting the outer and inner osteon. The load-induced nature of the fluid flow attributes to a long chain of lacunae and canaliculi also a large source of liquid which has to be transported through the network. This feature of acting as a source of fluid, which has to be drained via the network, causes these high velocities of the interstitial bone fluid. The higher fluid flow in osteon-in-osteons compared to ordinary osteons is a robust result that remains unchanged when considering an upper limit in pressure (Fig. 7).

This interpretation of the high fluid velocities found in osteon-in-osteons explains the high correlation between the mean fluid velocity and a structural parameter of the network: the average shortest path length. In particular, from the observation it becomes comprehensible that the relationships are opposite depending on the modeling approach (Fig. 6b). Using approach 1, a large shortest path length means long connecting paths between the cement line and the Haversian canal, and due to the fixed applied pressure, this long path causes high fluid resistance (i.e., low intrinsic permeability) and low fluid velocity. Consequently, fluid flow velocity and shortest path length are inversely proportional (i.e., higher tortuosity leads to lower intrinsic permeability). In approach 2 the source character of the LCN porosity implies longer paths have more fluid that must be drained into the Haversian canal. This can happen only by speeding up the fluid flow, and therefore, the average fluid flow velocity shows direct proportionality with the average shortest path length.

Figure 7 is key in our interpretation of the mechanobiological results. It shows which percentage of the canaliculi in the different types of osteons is stimulated by a shear force larger than the specific value given on the x-axis. For the range of shear forces that have been reported to elicit osteogenic responses in osteocyte-like cells through in vitro studies (Bakker et al. 2001; Smalt et al. 1997), the cumulative probability distributions are very similar for ordinary osteons and osteon-in-osteons. However, the conclusion that osteon type does not matter for mechanosensation is premature for several reasons. (i) It is unclear whether osteocytes could be “overstimulated,” i.e., their mechanosensitivity would again decrease for increasing shear forces. In vitro experiments investigating this have been performed in an artificial setting with cells adhering to planar surfaces. Progress has been made by performing such experiments with osteocyte-like cells (Lu et al. 2012). Using a genetically encoded fluorescent calcium indicator, it was recently shown in living mice that calcium spikes in osteocytes could be elicited for specific bending strains and frequencies, but that the intensity of the cellular response did not change when the load was further increased (Lewis et al. 2017). (ii) A big unknown in the interpretation of Fig. 7 is how the signals of individual osteocytes are integrated to an effective signal able to control osteoblast and osteoclast action on the bone surfaces (Cowin 2001). It was argued that osteocytes closer to the bone surface should contribute more to the integral signal (Mullender and Huiskes 1997). An alternative hypothesis to the averaging of signals from all osteocytes close to the surface is to consider only the largest signals (Hartmann et al. 2011). (iii) Like in most mechanobiological models, some of the input parameters (Table 1) are very challenging to measure and are, therefore, not sufficiently well characterized. As an example, the structure of the fibrous matrix in the pericellular space is not sufficiently known and, as a consequence, the value of the permeability of the pericellular space described by the parameter *k*_*p*,eff_ has some uncertainty (Sansalone et al. 2013). Given that *k*_*p*,eff_ occurs as a proportionality factor between the pressure difference and the average velocity in the canaliculus, effects are linear, i.e., a 10% increase in *k*_*p*,eff_ would cause a 10% decrease in the fluid pressure in approach 2. Based on reports on canaliculi crossing the cement line (Curtis et al. 1985), in particular, in younger individuals (Milovanovic et al. 2013), the influence of a “leaking” cement line was systematically studied (see Supplementary Material). An approximately linear effect was detected between the amount of fluid leaking through the cement line and the average pressure and fluid flow, while the characteristic pressure and flow patterns of the osteons types were maintained. (iv) Most importantly, the shear stresses are very similar for the two osteon types only for the chosen strain rate of $\dot{\epsilon }$ = 0.015 s^−1^, which corresponds to vigorous exercise like running. For more everyday physical activities like walking, the corresponding strain rate is fivefold lower (Al Nazer et al. 2012; Lanyon et al. 1975; Milgrom et al. 2002). For comparison, the dashed line in Fig. 7 represents the cumulative probability distributions for this more moderate loading of bone. In the case of walking and other physical activities of even lower loading, the large majority of osteocyte processes in ordinary osteons would not receive sufficient stimulation to elicit an osteogenic response. In osteon-in-osteons the fluid flow through roughly 31% of the canaliculi is still fast enough to overcome the stimulation threshold for osteocytic mechanotransduction. For moderate loading the strain amplification mechanism via load-induced fluid flow is more efficient in osteon-in-osteons. Considering that the frequency of osteon-in-osteons increases with age (Andreasen et al. 2018; Ericksen 1991; Yoshino et al. 1994), it can be speculated that this formation of more osteon-in-osteons is a potential mechanism to compensate for a general decrease in mechanosensation.

The possibility to assess the fluid flow in macroscopic samples of bone opens a new path to test the fluid flow hypothesis of bone’s mechanosensitivity. Our approach can be employed at a larger tissue level, complementing attempts in decoding the molecular mechanisms which function to transduce the interstitial fluid flow into a biochemical signal. To do so the strategy would be to use in vivo models to connect the information of bone formation and resorption sites obtained by in vivo micro-CT (Birkhold et al. 2015; Lambers et al. 2015) or labeling techniques (Carrieroa et al. 2018) under specified loading conditions. This information about the load-induced remodeling response could then be spatially correlated with models of the fluid flow through the canalicular network.

## Electronic supplementary material

Below is the link to the electronic supplementary material.

Supplementary material 1 (PDF 945 kb)
