# Supplementary material for: Network architecture strongly influences the fluid flow pattern through the lacunocanalicular network in human osteons
Source: Biomech Model Mechanobiol. 2019 Nov 28;19(3):823–40. doi: 10.1007/s10237-019-01250-1 (PMC7203595; doi:10.1007/s10237-019-01250-1)
Supplement: Supplementary file 1 — Supplementary material 1 (PDF 945 kb) [file 10237_2019_1250_MOESM1_ESM.pdf]

Supplementary Material

Saturation pressure

To study the robustness of approach 2, the volumetric fluid flow sources ($\boldsymbol{f}$ in eq. 14) of the osteons were limited by utilizing non-ideal Norton's equivalent current sources (Johnson 2003). In these sources the fluid output is limited as the fluid pressure reaches a defined saturation pressure, to take into consideration the compressibility of the interstitial bone fluid under high pressures. Limiting conductors with conductivity $\boldsymbol{C_{lim}}$ were introduced in parallel with the ideal volumetric flow sources f_i in each node i. As a result the limited volumetric flow $\boldsymbol{f_{lim}}$ goes to zero when the pressure $\boldsymbol{p}$ approaches the saturation pressure p_{max} ,

$$C_{lim,i} = f_i / p_{max} \quad (15)$$

and, therefore,

$$f_{lim,i} = f_i - p_i \cdot C_{lim,i} = f_i \left(1 - \frac{p_i}{p_{max}}\right). \quad (16)$$

The maximum possible pressure in the osteon is thus equal to the saturation pressure. Due to the already lower values of the pore pressures occurring in the normal osteon, the choice of the saturation pressure has hardly any effect on the average fluid velocity when chosen above 1000 kPa (Fig. 8a). The maximum possible pressure in human osteons is estimated to be 12-18% of the applied stress (Weinbaum et al. 1994; Zhang et al. 1998). Under stresses occurring during exercise the saturation pressure is, therefore, likely to be above 5000 kPa (Al Nazer et al. 2012; Jonkers et al. 2008). For a saturation pressure of 5000 kPa this would result in a reduction of the average fluid flow velocity by 11% for osteon-in-osteons. Taking for comparison the osteon-in-osteon with the largest values of pore pressure, the fluid flow and pressure patterns are still qualitatively very similar (Fig. 8b). The resulting pressure pattern obtained for a saturation pressure of 5000 kPa is indistinguishable from the one shown in Figure 3d.

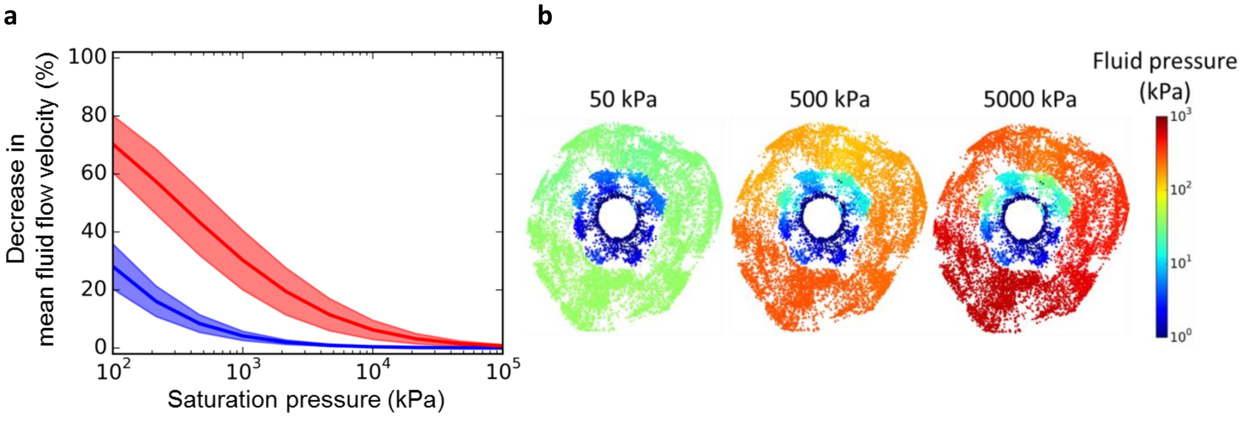

Figure 8. The effect of a pressure limitation on the average fluid flow velocity in normal osteons and osteon-in-osteons. a) The relative reduction of the average fluid flow velocity is plotted as function of the saturation pressure. The shaded region denotes the standard deviation. b) The pressure pattern with saturation pressures of 50 kPa, 500 kPa and 5000 kPa, respectively.

Leaking cement line

In our study we modelled the cement line as an impermeable boundary. To study the sensitivity of the model to a leaking cement line, a total leakage flow was added to the volumetric fluid flow sources f (see eq. 14) of only the cement line nodes:

$$f_i = f_i + \frac{\text{total cement line leakage}}{\text{number of cement line nodes}} \quad (17)$$

Since the direction of the flow at the cement line is unknown we simulated both possibilities, i.e. leakage into and out of the osteon (positive and negative leakage, respectively). The volumetric leakage flow was chosen to be equal to a percentage of the total volumetric flow within the Haversian canal of the osteon. For example, with a leakage of 5% and a total volumetric flow into the Haversian canal of 100 $\mu m^3/s$, the 'total cement line leakage' would be 5 $\mu m^3/s$. Both osteon types keep their characteristic pressure patterns in these simulations, as demonstrated by the pressure plots in figure 9a. An approximately linear effect was detected between the amount of fluid leaking through the cement line and the average pressure and fluid flow (fig. 9b). Since realistic values of leakage are likely below 1%, the consideration of a leaking cement line does not change our conclusions concerning the difference between normal osteons and osteon-in-osteons.

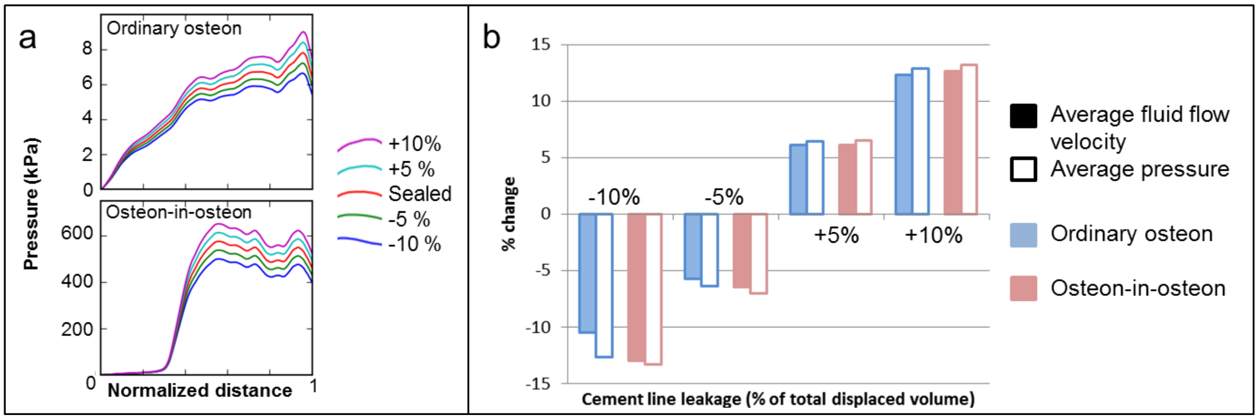

Figure 9. Consideration of a leaking cement line in the model a) Pressure profiles plotted as function of the normalized distance. As in figure 4 in the manuscript the distance is normalized so that Haversian canal = 0, cement line = 1. Four different cases of cement line leakage (all severe cases of leakage) were compared to the impermeable cement line (red line). b) The relative impact of the four leakage scenarios on average fluid flow velocity and pressure in the two representative osteons from figure 2 from our study, quantified as %change from the reference case of an impermeable cement line.

References

Al Nazer R, Lanovaz J, Kawalilak C, Johnston JD, Kontulainen S (2012) Direct in vivo strain measurements in human bone-A systematic literature review J Biomech 45:27-40 doi:10.1016/j.jbiomech.2011.08.004

Johnson DH (2003) Origins of the equivalent circuit concept: The current-source equivalent P Ieee 91:817-821 doi:10.1109/Jproc.2003.811795

Jonkers I, Sauwen N, Lenaerts G, Mulier M, Van der Perre G, Jaecques S (2008) Relation between subject-specific hip joint loading, stress distribution in the proximal femur and bone mineral density changes after total hip replacement J Biomech 41:3405-3413 doi:10.1016/j.jbiomech.2008.09.011

Weinbaum S, Cowin SC, Zeng Y (1994) A Model for the Excitation of Osteocytes by Mechanical Loading-Induced Bone Fluid Shear Stresses J Biomech 27:339-360 doi:Doi 10.1016/0021-9290(94)90010-8

Zhang D, Weinbaum S, Cowin SC (1998) Estimates of the peak pressures in bone pore water J Biomech Eng-T Asme 120:697-703 doi:10.1115/1.2834881
